# Extraplaque Laser to Assist in Crossing Occlusion (EL TACO): A Novel Method for Uncrossable Lesions

**DOI:** 10.1016/j.jscai.2023.100983

**Published:** 2023-05-04

**Authors:** Jarrod D. Frizzell, Brett L. Wanamaker, James A. Kong

**Affiliations:** a Heart and Vascular Institute, The Christ Hospital, Cincinnati, Ohio; b Division of Cardiovascular Medicine, Frankel Cardiovascular Center, University of Michigan, Ann Arbor, Michigan

**Keywords:** atherectomy, chronic total occlusion, percutaneous coronary intervention, uncrossable lesion

Uncrossable coronary lesions are those that have been successfully traversed with a guide wire into the distal lumen but are unable to be crossed with a balloon or other equipment for successful percutaneous coronary interventions (PCIs). These are commonly encountered in chronic total occlusions (CTOs), representing up to 9% of such lesions.^1^ Published algorithms have delineated systematic approaches for solving uncrossable lesions to improve PCI success.^2^^,^^3^ Methods described include increasing guide support, employing smaller balloons, balloon-assisted microdissection, alternative microcatheters (stiffer or smaller profile), atherectomy (laser or rotational), or hydraulic fracturing with microcatheter injections. A subset of approaches involves introducing a second wire into the subintimal space for balloon anchoring^3^ or external plaque modification via an “external cap crush.”^2^^,^^3^ In this latter technique, an inflated balloon within the subintimal space surrounding the uncrossable lesion modifies the resistant area such that equipment may pass over the luminal wire. Although laser atherectomy has been described in uncrossable algorithms, and its use has previously been safely demonstrated in the subintimal space as part of CTO PCI,^4^ here we describe the use of extraplaque laser to assist in crossing occlusions, a novel technique for uncrossable lesions.

## Case report

**Figure 1:**
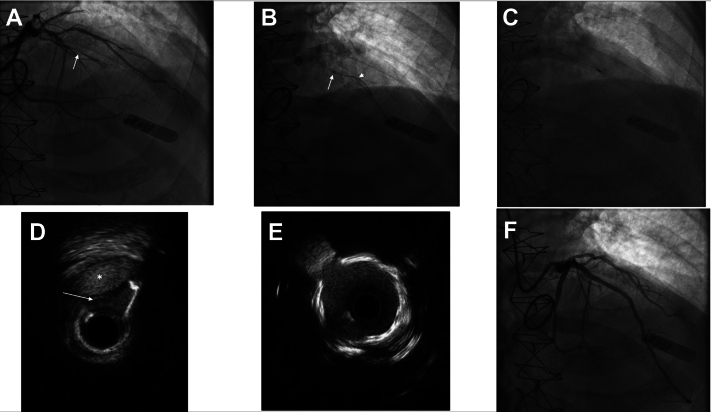
**Extraplaque laser to assist in crossing occlusionsstepwise approach.** (**A**) Chronic total occlusion (CTO) of mid left anterior descending (arrow) with bridging collateral providing distal flow in conjunction with retrograde collaterals (not shown). (**B**) A 0.9-mm Excimer laser (Philips) shown at the point of subintimal entry over the Gladius Mongo wire (Asahi Intecc), with continued ablation until just distal to the CTO segment (arrowhead). (**C**) A 1.5-mm rotational atherectomy burr (ROTAPRO, Boston Scientific) taken through the lesion. (**D**) Intravascular ultrasound following the extraplaque laser to assist in crossing occlusion method with subsequent rotational atherectomy showing subintimal space used in extraplaque laser to assist in crossing occlusion (asterisk) and disruption of circumferential calcification adjacent to the dissection plane (arrow). (**E**) Post–percutaneous coronary intervention intravascular ultrasound at the site of CTO showing full stent expansion and apposition. (**F**) Angiography after percutaneous coronary intervention showing no residual lesion.

A 76-year-old man with a history of aortic stenosis with prior mechanical aortic valve replacement, hypertension, and hyperlipidemia had new anginal symptoms that were initially managed with empiric antianginals. He later had a separate syndrome that led to the diagnosis of esophageal cancer. Cardiac catheterization for persistent angina showed critical lesions in the right coronary and left circumflex arteries, as well as a short CTO of the mid left anterior descending coronary artery (LAD) (Figure 1A). A multidisciplinary heart team considered him ineligible for coronary artery bypass grafting due to redo sternotomy, age, and active cancer and recommended complete revascularization by PCI. After successful PCI of his right coronary and left circumflex arteries, there was a failed attempt at LAD CTO PCI. He began chemotherapy and radiation treatment for his cancer. In the weeks following, he was admitted on 3 occasions with unstable anginal symptoms and found to have non–ST-elevation myocardial infarctions each time. Although his antianginal regimen was increased to the point where he was on maximally tolerated doses of 3 different agents (metoprolol, isosorbide mononitrate, and ranolazine), he continued to have symptoms. The addition of a calcium channel blocker was limited by hypotension. Because of continued symptoms and prior failed PCI attempt, he was transferred during the next admission to a high-volume CTO PCI center.

After use of Fielder XT-A (Asahi Intecc), Gladius Mongo (Asahi Intecc), Pilot 200 (Abbott), Gladius (Asahi Intecc), and Gaia Next 3 (Asahi Intecc) wires, the lesion was crossed with a Sion Black (Asahi Intecc) supported by a Corsair Pro XS microcatheter (Asahi Intecc); however, the microcatheter was unable to cross the lesion. The following methods were attempted using published algorithms.^2^^,^^3^ A 1.5-mm Takeru balloon (Terumo) would not advance, including far enough for effective balloon-assisted microdissection. A 0.9-mm Excimer laser (Philips) would not cross the lesion at the highest settings (fluency, 80 mJ/mm^2^; frequency, 80 Hz). Attempting to exchange the Sion Black for a Rotadrive wire (Boston Scientific) in anticipation of rotational atherectomy was unsuccessful. Last, a dual-lumen microcatheter (Sasuke, Asashi Intecc) was used to introduce a Gladius Mongo wire, which was subsequently knuckled into the subintimal space around the lesion. An external cap crush over the subintimal wire was unsuccessful, as even a 1.5-mm balloon would not pass.

Next, the laser was taken over the Mongo wire, where it initially stopped at the point of subintimal entry (Figure 1B). While engaging at fluency and frequency settings of 80/80, gentle pressure was applied that led to advancement of the laser into the dissection plane surrounding the CTO segment, and it was stopped in the subintimal plane just distal to the occlusion. Flushing with saline or contrast was not performed for 2 reasons. First, because the lesion was uncrossable, the laser may act as a “plug” obstructing flow, leading to increasing pressure in the proximal vessel with risk of hydraulic dissection and proximal extension, jeopardizing the large diagonal and other vessels. Second, if the laser was as obstructive to flow as presumed, then the focal photoablative effect would predominate. Reduced flow around the laser may avoid the usual cavitation of the medium (saline or contrast) with less photomechanical effect, thus decreasing the risk of perforation or expansion of subintimal hematoma. Following use of the laser, the microcatheter was advanced through the lesion and Sion Black exchanged for a Rotadrive wire, over which rotational atherectomy was performed with a 1.5-mm burr (ROTAPRO, Boston Scientific) (Figure 1C). Intravascular ultrasound at the CTO segment showed focal disruption of circumferential calcium adjacent to the dissection plane (Figure 1D) corresponding to the extraplaque laser modification of the CTO segment. Overlapping 3.5 mm × 38 mm and 2.5 mm × 38 mm drug-eluting stents were placed from the LAD ostium through the mid vessel, postdilated proximally with a 4.5-mm noncompliant balloon and in the midsection with a 3.5-mm noncompliant balloon. Intravascular ultrasound showed full stent expansion and apposition (Figure 1E) with no residual lesion by angiography (Figure 1F).

## Discussion

Extraplaque laser to assist in crossing occlusions is a novel method to assist in successful PCI for uncrossable lesions and may serve as a useful adjunct to previously published algorithms.
